# 3D printed microfluidic lab-on-a-chip device for fiber-based dual beam optical manipulation

**DOI:** 10.1038/s41598-021-93205-9

**Published:** 2021-07-16

**Authors:** Haoran Wang, Anton Enders, John-Alexander Preuss, Janina Bahnemann, Alexander Heisterkamp, Maria Leilani Torres-Mapa

**Affiliations:** 1 Institute of Quantum Optics, Gottfried Wilhelm Leibniz University Hannover, Welfengarten 1, 30167 Hannover, Germany; 2 Lower Saxony Centre for Biomedical Engineering, Implant Research and Development (NIFE), Stadtfelddamm 34, 30625 Hannover, Germany; 3 Institute of Technical Chemistry, Gottfried Wilhelm Leibniz University Hannover, Callinstrasse 5, 30167 Hannover, Germany

**Keywords:** Optical manipulation and tweezers, Fluidics

## Abstract

3D printing of microfluidic lab-on-a-chip devices enables rapid prototyping of robust and complex structures. In this work, we designed and fabricated a 3D printed lab-on-a-chip device for fiber-based dual beam optical manipulation. The final 3D printed chip offers three key features, such as (1) an optimized fiber channel design for precise alignment of optical fibers, (2) an optically clear window to visualize the trapping region, and (3) a sample channel which facilitates hydrodynamic focusing of samples. A square zig–zag structure incorporated in the sample channel increases the number of particles at the trapping site and focuses the cells and particles during experiments when operating the chip at low Reynolds number. To evaluate the performance of the device for optical manipulation, we implemented on-chip, fiber-based optical trapping of different-sized microscopic particles and performed trap stiffness measurements. In addition, optical stretching of MCF-7 cells was successfully accomplished for the purpose of studying the effects of a cytochalasin metabolite, pyrichalasin H, on cell elasticity. We observed distinct changes in the deformability of single cells treated with pyrichalasin H compared to untreated cells. These results demonstrate that 3D printed microfluidic lab-on-a-chip devices offer a cost-effective and customizable platform for applications in optical manipulation.

## Introduction

Microfluidic lab-on-a-chip (LOC) requires highly precise, fine structures in order to transport and manipulate fluids in a targeted manner. LOCs are characterized with micrometer-sized channels which facilitate laminar fluid flow, where conditions are governed by viscous forces and inertia can be considered negligible. To achieve this, LOC devices are produced conventionally via soft lithography using poly(dimethylsiloxane) (PDMS) as the material, which has been the workhorse in the development of microfluidic technology. The fabrication steps in soft lithography include producing a mold in a clean room environment and complex chemical etching processes that necessitates substantial expertise^1,2^. Other techniques such as injection molding, micromilling and hot embossing have been also employed as microfabrication methods^3^ with the aim to address high-throughput manufacturing and low cost. These alternative approaches can similarly produce channels with dimensions of tens to hundreds of micrometers, each technique having its own unique advantages and disadvantages. However, for microfluidic technology to be reproducible and truly accessible to the end-users, methods that enable rational design and predictable performance are highly desirable^4^.

3D printing has recently emerged as a rapid, single-step approach to fabricate LOC devices^1,4^. 3D printing produces cost-effective microfluidic devices and generates high resolution, complex 3D structures compatible with materials of various mechanical and optical properties. Using this technique, a microfluidic device is designed with computer-aided design (CAD) software and then printed out layer by layer^4^. Compared to other approaches, 3D printing is relatively straightforward, requires minimal post-processing and avoids critical error-prone bonding steps. Furthermore, 3D printing also enables parallel prototyping—a crucial feature which can substantially reduce the time lag between the initial design and the creation of a finished and functioning prototype^5^. Recent progress in 3D printing and the increased availability of commercial 3D printers have enabled the use of this technology for various applications. 3D printing has been used to fabricate LOC devices, e.g., to evaluate various microfluidic mixer designs^5^ and perform particle analysis^6^. In addition, modular 3D printed platforms with pneumatic control have permitted the generation of emulsion containing droplets^7^. These exemplary studies show the versatility of 3D printing for various microfluidic LOC applications.

Our aim in this work was to fabricate a 3D printed microfluidic LOC device for optical manipulation experiments. Optical manipulation has been used for position control, precise measurements of forces in biological samples and as a non-contact method to probe viscoelastic material properties. The microfluidic LOC environment offers continuous delivery of samples at the trapping region and a well-defined fluidic environment while maintaining sample sterility, which is particularly important for cell culture applications. Dual beam fiber-based optical trap is the most common geometry integrated in microfluidic LOC for optical manipulation. In this experimental configuration, two coaxially aligned optical fibers deliver counterpropragating laser beams to capture microscopic particles ^27^or living cells ^28^. As a result of the divergent nature of the laser beams, the laser intensity at the sample is lower compared to single beam optical traps. Fiber-based optical traps are compatible with microfluidic LOCs—creating compact and economical platforms well-suited for optical manipulation. Consequently, they have been explored in various configurations, including in applications of optical chromatography^8^, cell sorting^9–12^ and high-throughput optical targeting of mammalian cells for intracellular dye delivery^13,14^. Furthermore, in combination with fluorescence or spectroscopy techniques, cancer cells can be be identified and sorted based on their unique molecular fingerprint^15^, the relative levels of specific molecules present in bacteria spores can be quantified^16^ and infectious virions can be studied in their native environment^17^.

Due to the wide variety of applications of microfluidic LOC in optical manipulation, several fabrication methods have been implemented in order to improve the integration of these techniques. For example, polymer chips for optical manipulation were assembled using multi-step mold fabrication via hot embossing^18^ and injection molding^19,20^. Similarly, femtosecond micromachining was employed to fabricate readily aligned waveguides and channels in a glass chip for optical stretching^21,22^ and sorting^23,24^ of cells. Despite these technological advances, however, problems with complex fabrication still persist, largely stemming from multi-step procedures, requiring lengthy and stringent methods in order to produce a working prototype.

In this work, we demonstrate a fully characterized microfluidic LOC device for dual beam optical fiber trapping using 3D printing technology. The device was fabricated in a one-step procedure including both inlet and outlet ports for tubings, as well as 3D channels, a square zig–zag section and fiber channels for fiber insertion. We outline the 3D printing steps and channel design considerations that were deployed. To validate the applicability of the chip for optical manipulation, we implemented on-chip dual beam fiber-based optical trap and characterized the system’s trapping performance. Using the same microfluidic LOC device, we successfully achieved optical stretching of cancer cells in the presence of a metabolite to determine its effect on a cell’s viscoelastic properties. Overall, this work highlights the great potential of 3D printing customized LOC devices for precise optical manipulation and future potential applications of this promising technology in colloidal and single-cell biological studies.

## Results

### Calibration of the printing resolution of the 3D printer

**Figure 1:**
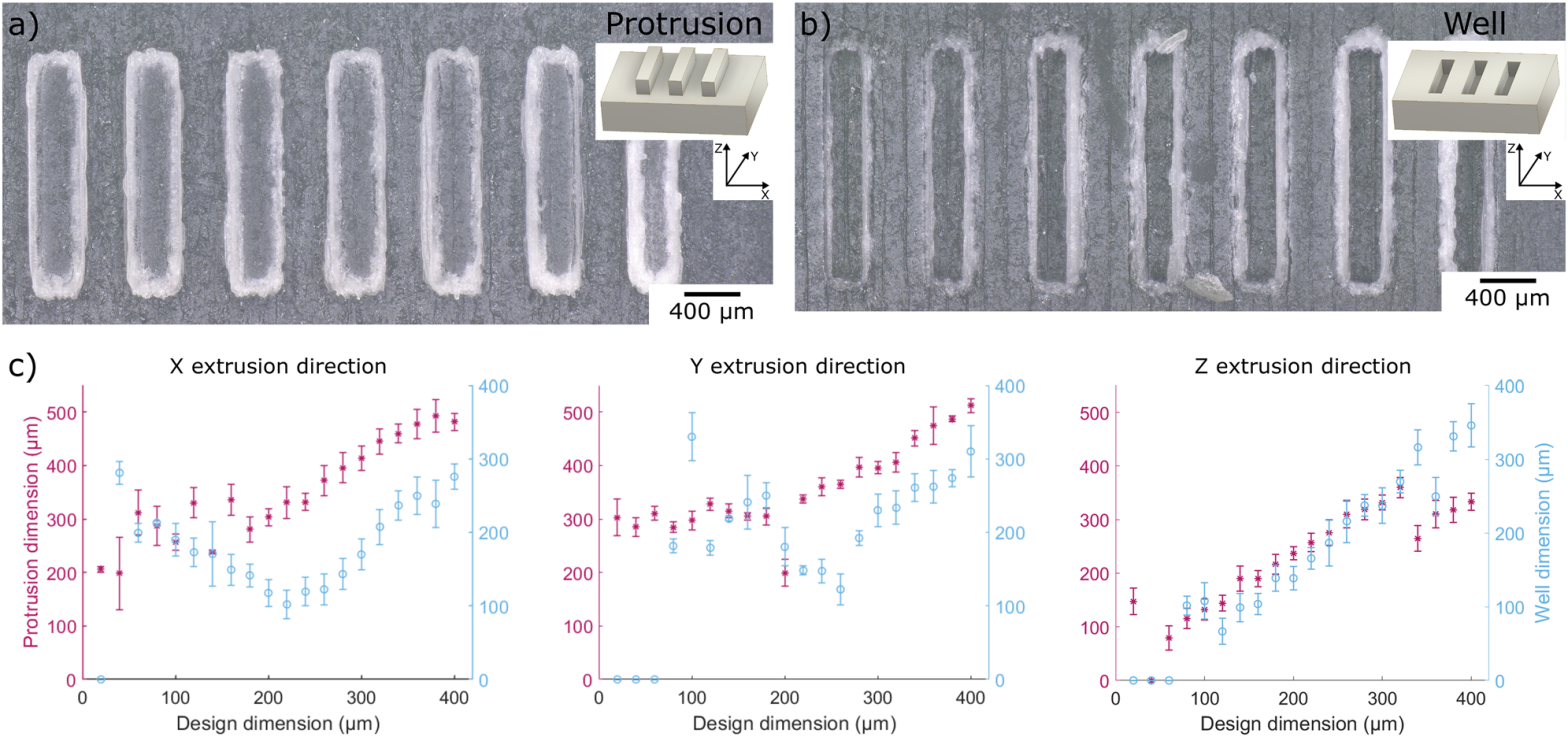
Calibration of the printing resolution of the 3D printer. Test structures were designed using a CAD software and 3D printed using Projet MJP2500 Plus Multijet printer. Example microscope images of the 3D printed periodic structures such as (**a**) protrusions and (**b**) wells with a designed width of $360\,\upmu \mathrm{m}$. Insets show the schematic of the structures and the extrusion directions. (**c**) The dimensions of the 3D printed target features were measured and plotted as a function of the design dimension for the three different extrusion directions. Error bars correspond to $\pm 1$ standard deviation.

The high-definition Projet MJP2500 Plus Multijet printer (3D Systems, Rock Hill, USA) was used to fabricate the microfluidic devices. In order to characterize the printing resolution of the 3D printer, we measured the 3D printed feature as a function of the design dimension. For this purpose, a test structure with rectangular-shaped wells and protrusions of different widths ranging from 20 to $400 \,\upmu \mathrm{m}$ with a length of 2 mm and a height or depth of 0.5 mm were designed using CAD as shown in Fig. 1a,b. For each width, 10 protrusions and wells were measured. After 3D printing and post-processing, the 3D printed test structures were imaged under a digital 3D microscope and the dimensions were measured at approximately half the height of the protrusion or well. Figure 1c shows the corresponding measurements of the features printed in X, Y, Z extrusion directions. It can be seen that the accuracy of the 3D printed test structure vary depending on the shape (protrusion or well) and the extrusion direction it is printed. For protrusions, the minimum printable features are approximately $200\,\upmu \mathrm{m}$, $200\,\upmu \mathrm{m}$ and $100\,\upmu \mathrm{m}$ whereas wells can be printed at smaller dimensions at approximately $100\,\upmu \mathrm{m}$, $200\,\upmu \mathrm{m}$ and $100\,\upmu \mathrm{m}$ for X, Y, and Z extrusion directions respectively. Especially when printing along the X and Y, smaller dimensions less than the minimum printable feature design are not accurately reproduced. In comparison, the printed dimensions are linearly correlated to the design dimension when printing along the Z direction. For designs larger than the minimum printable feature, the 3D printed test structures depict the design dimensions more accurately. The surface roughness can also be easily seen on the microscopes images of the 3D printed structures as shown in Fig. 1a,b. The channel walls of both 3D printed wells and protrusions exhibit large surface roughness. Surface roughness (arithmetic average of roughness) are determined to be approximately $9\,\upmu \mathrm{m}$, $9\,\upmu \mathrm{m}$, $2\,\upmu \mathrm{m}$ for X, Y, Z extrusion directions, respectively.

### Design and fabrication of the 3D printed microfluidic device

**Figure 2:**
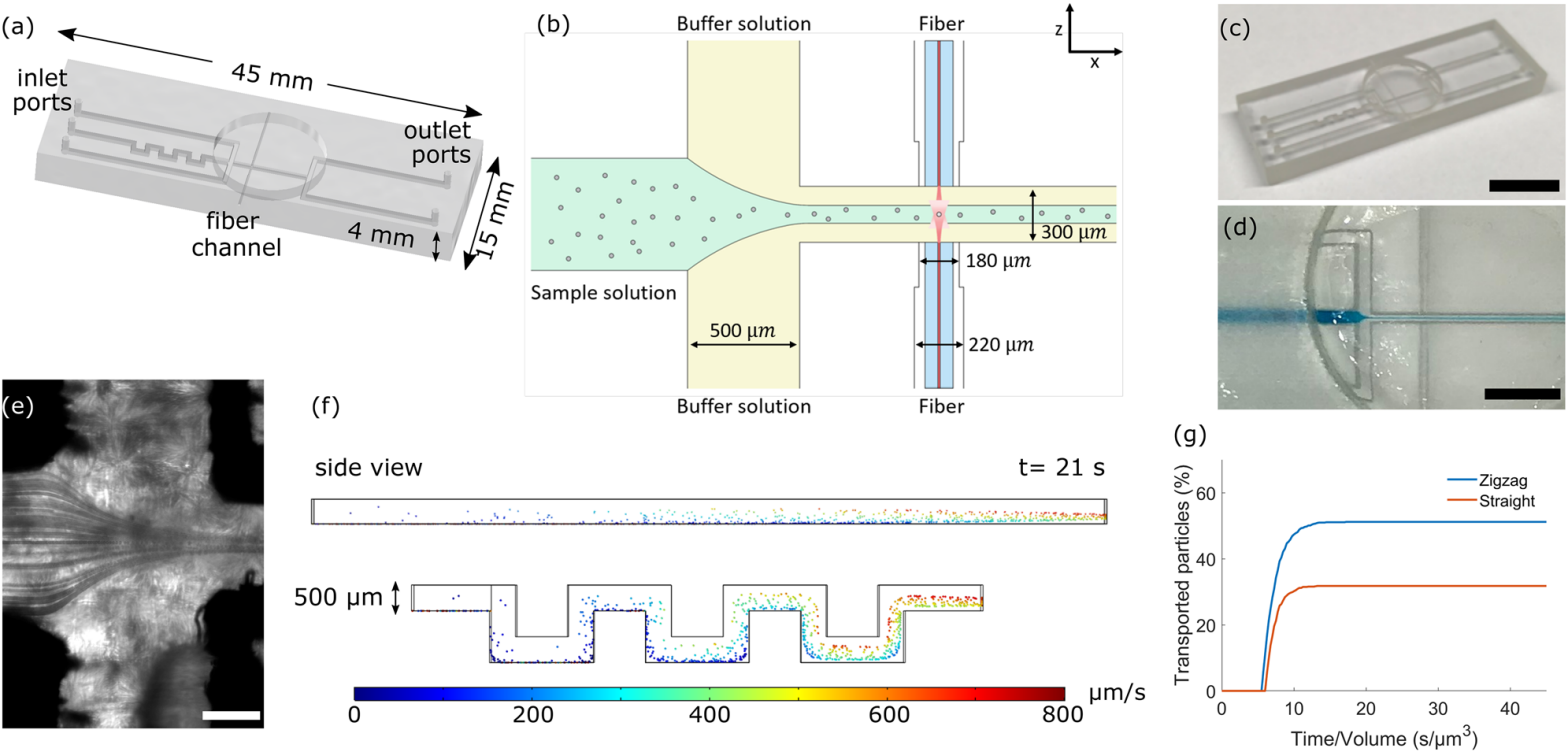
Design and proof-of-concept of the 3D printed microfluidic chip. (**a**) The 3D design of the microfluidic chip with the corresponding dimensions. (**b**) A schematic illustration of the principle of the dual beam optical trap system in the designed 3D printed microfluidic chip is shown. The sample solution is hydrodynamically focused and sheathed by the buffer solution. (**c**) An exemplar photo of the 3D printed microfluidic chip. Scale bar is 10 mm. (**d**) Water containing color additive is flown through the chip to simulate the sample solution. Scale bar is 4 mm.  (**e**) Trajectory of $10 \,\upmu \mathrm{m}$ polystyrene particles flowed through the sample channel. Scale bar is 200 µm. COMSOL simulations are presented in (**f**) showing the speed of the transported particles for a straight (top) and square zig–zag mixer (bottom) structure. The percent transported particles at the end of zig–zag and straight channels with the same total path length is shown in (**g**) as a function of time/volume.

The microfluidic device was designed using a commercially available CAD software, Inventor 2019 (Autodesk, Version 2019, California, USA). Figure 2a shows the chip design and overall dimensions. The chip has three square-shaped inlets with a cross-sectional width of $500\,\upmu \mathrm{m}$ designed to flow the buffer and sample solutions (Fig. 2b). Samples containing cells or polystyrene particles were intended to flow through the middle channel, while the buffer solution was pumped through the outer two channels. To enhance the number of particles and cells reaching the trapping region at a time, one section of the middle channel contained a square zig–zag structure. Fluids from the three inlets converged and could hydrodynamically focus the particles and cells in the middle channel as they traverse the trapping region. In order to obtain high quality images, a circular opening was incorporated above the trapping region to accommodate a round glass cover slip. Two single mode optical fibers were inserted in the fiber channels, which were also directly embedded in the 3D printed chip. The initial portion of the fiber channels has a width of $220\,\upmu \mathrm{m}$ for smooth fiber insertion whereas the distal portion (which was directly connected to the flow channel) has a smaller width of $190 \,\upmu \mathrm{m}$ to confine the fiber. This design fixed the optical fibers within a small tolerance which improved the success of the manual fiber alignment. A photo of the final 3D printed chip is shown in Fig. 2c. All dimensional widths of the channels stated here are the designed dimensions in the CAD design.

To demonstrate hydrodynamic focusing, water mixed with blue color additive was flowed in the middle channel and water in the outer two channels (Fig. 2d). The focusing of the blue sample stream can be observed after the intersection of the sample (middle channel) and the buffer (upper and lower channels). We further confirmed hydrodynamic focusing by flowing $10\,\upmu \mathrm{m}$ particles in the sample channel with the sample and buffer flow speed set to $20\,\upmu \mathrm{l}/\mathrm{min}$ and $25\,\upmu \mathrm{l}/\mathrm{min}$, respectively. Figure 2e shows the trajectories of the particles as they flow through the middle channel of the LOC. After the intersection of the three microchannels, the particles are focused on a straight and narrow trajectory by the flanking of the buffer streams.

Computational fluid dynamics (CFD) simulation was performed to quantify the effect of the square zig–zag section to the fluid speed and the number of particles reaching the trapping site. We compared a straight and zig–zag channel with identical channel dimensions and total fluid path length (16 mm) at fluid speed ($\nu$) and Reynolds number (Re), ($\nu$=333 $\mu$m/s, Re=0.17) (Fig. 2f). The number of particles which reached the outlet was counted after a simulation time of 180 s for both straight and zig–zag structure. The additional structure resulted to more particles at the end of the zig–zag structure arriving at higher speeds compared to the straight channel (Fig. 2f). The incorporated square zig–zag structure increases the percentage of transported particles by about 50%, with more particles centered in the square channel, thus improving the throughput of particles at the trapping site (Fig. 2g). In comparison, for conditions with slow fluid flow speed and low Reynolds number, particles traveling in a long straight channel tend to disperse and sediment. The designed square zig–zag structure in the microfluidic chip minimizes sedimentation especially for larger particles and enables sufficient particles to reach the trapping site.

### Characterization of dual beam fiber optical trap

To validate the performance of the 3D printed microfluidic chip for optical manipulation, dual beam optical trapping of polystyrene particles was performed and characterized. Assuming that the same laser power was emitted from each fiber, a particle will be trapped at the equilibrium position, found in the middle of the two fibers, with the scattering forces arising from each beam being equal in magnitude but opposite in direction. To achieve successful trapping, the two fibers must be precisely aligned for particles to be trapped due to the combination of scattering forces from the coaxially aligned counter-propagating beams ^27^. Therefore, a crucial technical specification was that the fiber channels must enable fine positional control of the two fibers. Additionally, for any given laser power and particle size, the distance between the fibers affects the trapping stability. In our design, the width of the middle channel constrains the minimum fiber distance. Therefore, the performance of the trap was affected indirectly by the technical specifications of the 3D printer, taking also into account, the surface roughness of the channels and the ability to remove any residual support material from the microfluidic channel^25^.

**Figure 3:**
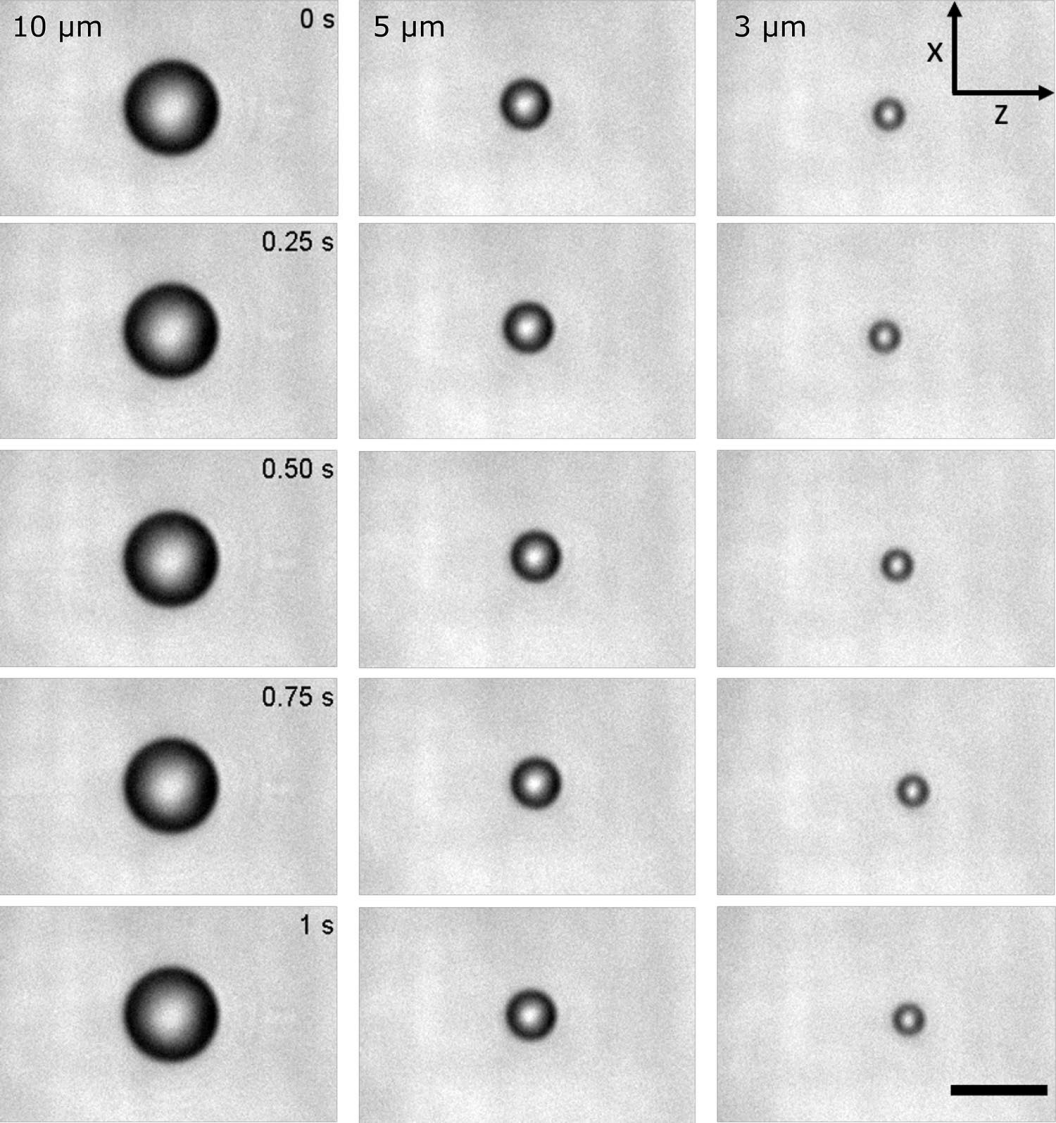
(Left to right) Images of $10\;\upmu\hbox {m}$, $5\;\upmu\hbox {m}$ and $3\;\upmu\hbox {m}$ optically trapped particles at different time points using laser power (*P*) = 200 mW from each optical fiber. Especially for $3\;\upmu\hbox {m}$ particle, position fluctuations within the 1 s recording duration can be observed. Scale bar is 10 µm.

To test our microfluidic device, water contanining low concentration of polystyrene particles of 1, 3, 5 and $10\;\mu \hbox {m}$ in size were flowed in the trapping region. First, the fluid speed was operated at $\nu$ $\approx$ 555 $\mu$m/s so that particles arrive at the trapping site. Once a single particle is trapped, the sample and buffer flow were set to a much lower speed ($\nu$ $\approx$ 30 $\mu$m/s). The  trapped particle was maintained in a weak trap for several minutes in order to stabilize the trap prior to measurements. For all experiments in this work, the particles or cells were captured by an optical trap under a very slow continuous flow. Figure 3 shows the video frames captured at different time points using a laser power (*P*) = 200 mW from each optical fiber. In most trapping experiments, the position of both 5 and $10\;\mu \hbox {m}$ particles were stable and localized within the middle of the trapping region. In contrast, the  trapped $3\;\mu \hbox {m}$ particle oscillated back and forth in the Z-direction, along the direction of laser propagation. $1\;\mu \hbox {m}$ particles escaped from the trapping region and cannot be captured with our dual beam optical trapping system.

**Figure 4:**
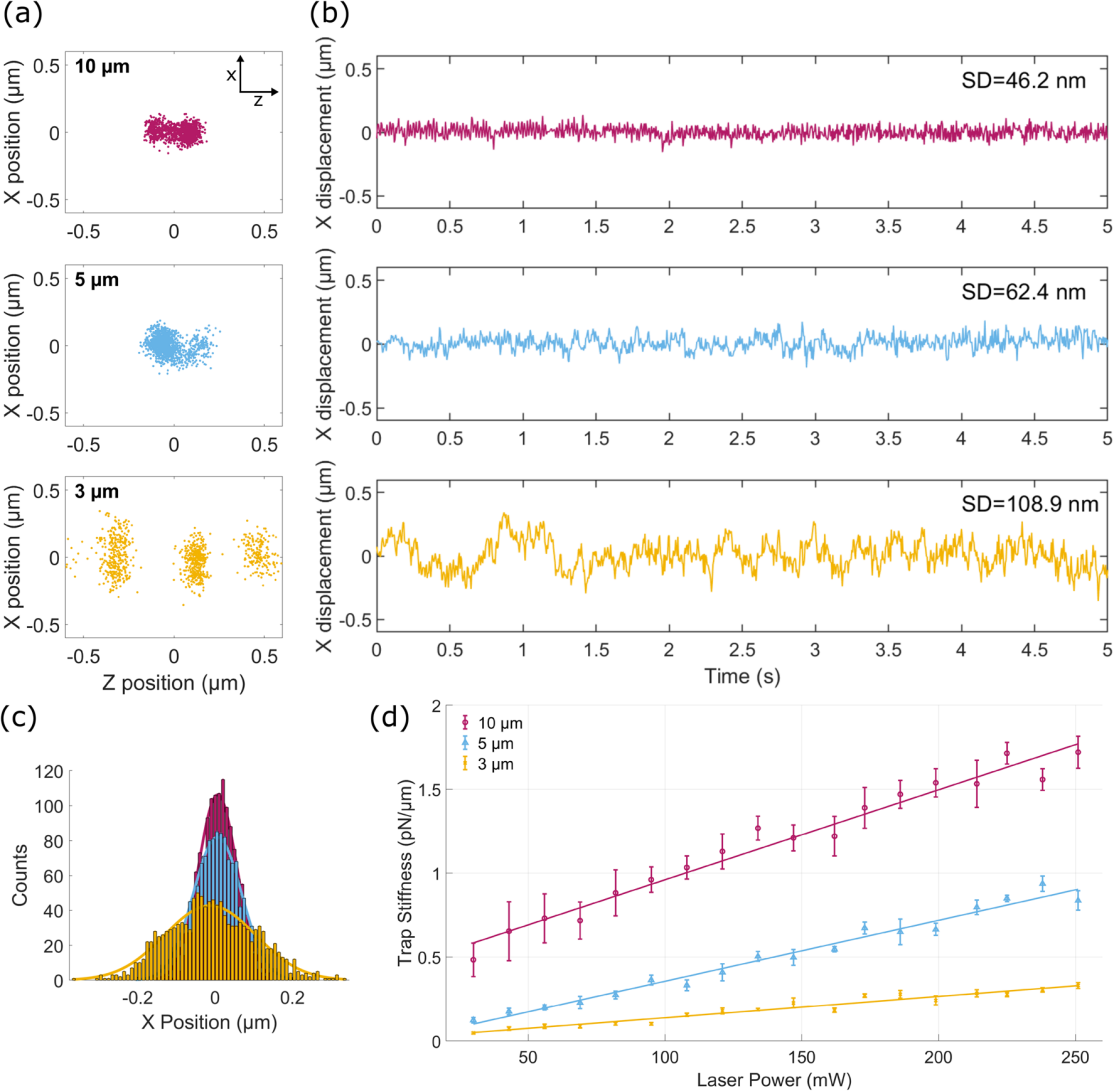
(**a**) Representative results for the X-Z position plot of the optically trapped particles at laser power, *P *= 240 mW from each beam. (**b**) Plot of the position fluctuation in X axis as a function of time. (**c**) Histogram of the position of the trapped particles at *P *= 240 mW. (**d**) Plot of the transverse trap stiffness for 3, 5 and $10\;\upmu\hbox {m}$ sized particles measured for different incident laser power from each fiber. Each data point is an average of n=5 trapped particles and error bars are $\pm 1$ standard deviation.

The time-varying position fluctuations of the trapped particles were analyzed at *P *= 240 mW for each fiber. 2D plots of the position (X vs. Z) show that 10 and $5\;\upmu \hbox {m}$ particles are stably trapped between the counterpropagating beams as shown in Fig. 4a. Meanwhile, the position of the trapped $3\;\mu \hbox {m}$ particle fluctuates at three different equidistant positions. The oscillation along the Z-direction observed for $3\;\mu \hbox {m}$ particle could indicate the presence of multiple stable trap positions^26^. The standard deviation (SD) of the time-varying trace in the X-direction is inversely correlated with the trapped particle sizes, 10, 5 and $3\;\mu \hbox {m}$, with SD = 46.2, 62.4 and 108.9 nm, respectively (Fig. 4b). Figure 4c shows the histogram of the X-position distribution for the three different sized particles. Based on the thermal equilibrium theory, the variance of the particle’s position distribution, $\sigma$ is related to the thermal energy of the particle, *k* $_B$*T* and the trap stiffness, $\kappa$ given by the equation,

1 $$\begin{aligned} \kappa =\frac{k_{B}T}{\langle(x-x_{0})^2\rangle} = \frac{k_{B}T}{\sigma ^2} \end{aligned}$$

where *k*$_B$ is the Boltzmann constant and *T* is the absolute temperature.

The transverse trap stiffness along the X-direction of 5 individually optically trapped particles was calculated and is shown in Fig. 4d. The trap stiffness has a linear dependence on the laser power for all particle sizes. At P= 250 mW exiting each fiber, the measured trap stiffnesses were ($1.72\pm 0.095$) pN/μm, $(0.85\pm 0.05)$ pN/μm and $(0.33\pm 0.017)$ pN/μm for 10, 5 and $3\;\mu \hbox {m}$ particles, respectively. In comparison to 5 and $3\;\mu \hbox {m}$ particles, optically trapped $10\;\mu \hbox {m}$ particles exhibited higher trap stiffnesses for all the laser powers which also explains their more restricted Brownian motion in the optical trap. For the given fiber separation distance and beam waist ($\approx$ 2.8 μm), trapping larger particle sizes resulted in a more stable transverse trapping efficiency compared to smaller particles^26^. At low laser powers and particles with similar dimensions to the beam waist, the trapped particle is sensitive to small perturbations which could also result to unexpected fluctuations in position.

### Deformability of cancer cells under an optical stretcher

To further demonstrate the capabilities of our 3D printed LOC, we employed the same device as an optical stretcher^28,29^. Cancer cells were stretched under increasing optical force to assess the potency of pyrichalasin H in affecting cell elasticity. Pyrichalasin H is a metabolite belonging to a family of fungal polyketide-amino acid hybrid molecules called cytochalasans, derived and isolated from rice blast fungus, Magnaporthe grisea NI980^30^. Cytochalasans are known to disrupt actin polymerization in mammalian cells^31–33^. Actin-binding studies in cells have shown that pyrichalasin H, similar to other cytochalasans, targets the actin in the cytoskeleton^34^. In the present work, single MCF-7 cells were optically stretched in the presence and absence of pyrichalasin H, to observe whether the compound affects the cell elasticity.

**Figure 5:**
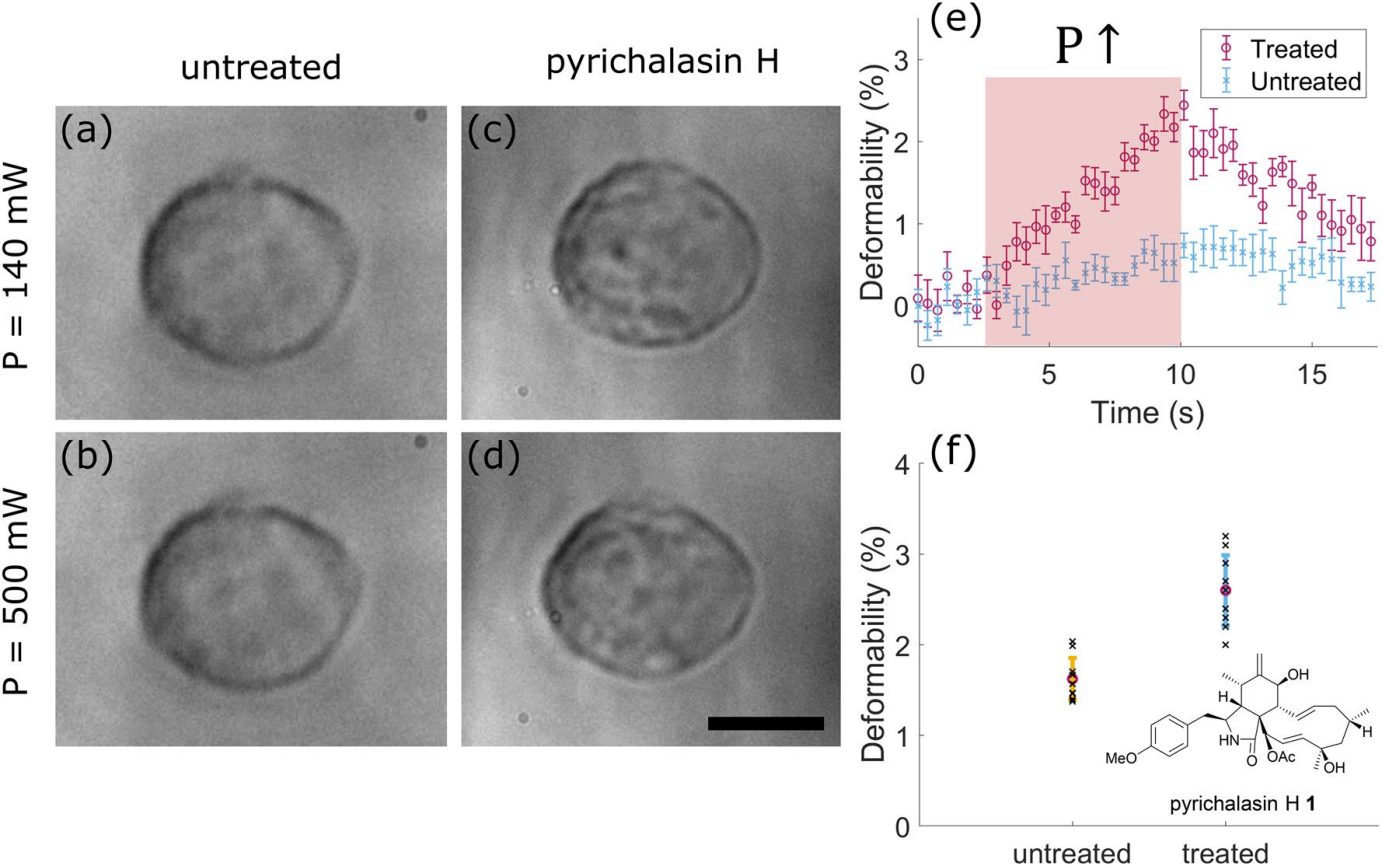
Example images of optical stretched breast cancer cells (MCF-7). The images in top row show untreated (**a**) and pyrichalasin H treated cells (**c**) at incident laser power (*P﻿*) of 140 mW from each fiber. The bottom row shows untreated (**b**) and treated (**d**) cells at *P *= 500 mW from each fiber. The treated MCF-7 cells show larger deformation at higher incident laser power. Scale bar is 10 μm. (**e**) Comparison of the deformability of untreated and treated MCF-7 cells with pyrichalasin H over time (n=5 cells). Error bars indicate $\pm 1$ standard error of the mean. The shaded area indicates when *P* is increased to 500 mW. High laser power (P = 500 mW) is applied for a fixed duration of 7 seconds. Afterwards, laser power is decreased to 140 mW. (**f**) Summary of the deformability for individual optically stretched cell. The average deformability is marked with a magenta circle (n=10 cells). Error bars correspond to $\pm 1$ standard deviation.

MCF-7 cells were first optically trapped at moderate laser power *P﻿ *= 140 mW and then the laser power was increased to *P﻿ *= 500 mW for 7 seconds to induce stretching, before being decreased once again to *P﻿ *=140 mW. The laser powers stated were measured from each fiber. The percentage change in cell length, which in this work is referred as deformability *d* was used to compare cell elasticity for each condition. To calculate *d*, the initial cell length ( $L_0$) at *P﻿ *=140 mW was used. Deformability *d* can therefore be calculated as follows,

2 $$\begin{aligned} d = \left( \frac{L-L_{0}}{L_{0}}\right) \times 100\% \end{aligned}$$

where *L* is the detected cell length for every frame of the video. MCF-7 cells treated and untreated with pyrichalasin H were optically stretched using the developed device. Figure 5a–d shows treated and untreated cells under trapped (*﻿P* = 140 mW) stretched (*P﻿* = 500 mW) conditions. It is evident that untreated cells have a lower deformability (up to 1%) compared to treated cells (up to 3%), which appear more elastic, especially at high laser power as shown in Fig. 5e. The maximum deformability of n=10 cells is summarized in Fig. 5f. The average deformability of untreated MCF-7 cells is $\mathrm{d}=(1.62\pm 0.23)\%$ or about 62% lower compared to treated cells $\mathrm{d}=(2.60\pm 0.39)\%$. Cell length changes fell in the range of 270-418 nm and 405-890 nm for untreated and treated cells, respectively. Because the resolution of our imaging system is 136 nm/pixel, most measured cell length fell above the Nyquist limit. Therefore, the results show that the treatment of cells with pyrichalasin H, at the concentration used for our experiments, indeed has an effect on the overall cell elasticity.

## Discussion

This paper demonstrates amply the promising potential of 3D printing technology to fabricate microfluidic chips that are suitable for optical manipulation experiments. Channel dimensions as small as hundreds of microns with acceptable roughness parameters can be produced with this technique. Those features render them adequate to maintain laminar flow during trapping experiments. 3D square zig–zag structures can also be incorporated into designs to facilitate increased particle speed when compared to straight channels. Notably, such a 3D square zig–zag structure cannot be produced with soft lithography without implementing multiple layer fabrication and bonding steps. With such structures, more particles and cells are transported at the trapping site and optically controlled using the dual beams from the optical fibers. Using our fabricated microfluidic chip, both optical trapping and cell stretching experiments can be performed in a reproducible manner due to the availability of samples at the trapping site. The 3D printing technology also enables multiple designs to be printed at the same time which speeds up the prototyping stage. A fully assembled chip takes about 4 h to fabricate including the printing ($\approx$ 2h), cleaning of the channels ($\approx$ 1h) and bonding of glass coverslip ($\approx$ 1h). A great advantage of the method is that the entire microfluidic chip including ports for inlet and outlets are readily incorporated in the design which minimizes bonding steps that could cause leakage and sample evaporation. Even with the additional bonding of the glass coverslip, experiments could be performed for several hours due to stability and robustness of the 3D printed microfluidic chip.

Several considerations must be taken into account when designing and printing the microfluidic chips. First, the overall thickness of the device is important to consider especially when cleaning the chip using heated oil as we have done in this work. For thin chips, heating may cause the material to soften and cause unwanted bending on the device. Our stable prototype with an overall thickness of 4 mm was able withstand the cleaning procedure using heated oil and maintain its structural integrity. Second, the direction of printing is also crucial to obtain high resolution structures. In order to fabricate high resolution channels, the relatively large fluid channels are aligned along the Y extrusion direction where as the fiber channels are oriented along the X extrusion direction. Third, the microchannel shapes might be limited depending on the minimum printable features of the 3D printer ($\approx$ 100 μm). V-shaped microchannels which are often used to confine optical fibers cannot be reliably fabricated at the appropriate dimensions. Hence, square-shaped microchannels were used in the design. Fourth, calibration of the 3D printed structures should be performed to be able to predict the final 3D printed dimension. This should also account for any surface roughness of the microchannel walls. Previous studies have measured the surface roughness for several models of 3D printers and compared their surface roughness. Overall, 3D printed structures can achieve sub-micrometer surface roughness outperforming techniques such as stereolithography and fused deposition modeling but still comparatively inferior to micromilling and two photon polymerization methods^35^. In our work, channel walls of the microfluidic channel exhibit considerable surface roughness, larger than those reported. Despite of the considerable surface roughness, controlled fluidic flow at low Reynolds number condition is still possible enabling us to perform sensitive optical trapping and stretching experiments.

One current limitation of the 3D printing technology is the optical transparency of the microchannels due to surface roughness of the cured materials. Optical manipulation experiments use microscopy to visualize the trapping process. The surface roughness and optical transparency of the cured material, often proprietary in nature, could potentially impede experiments that involve microscopy and spectroscopy^36^. Future materials with higher optical transparency upon curing (especially in the visible spectral wavelengths) and increase in 3D printing resolution would therefore be desirable for analytical and imaging experiments. Although post-processing methods could be performed such as sanding, polishing and smoothing on the outer surface of the microfluidic chip to improve surface roughness, the embedded microchannel surface roughness are difficult to reduce without resorting to chemical treatment. Our approach to bond a glass coverslip for image acquisition circumvented this problem and enabled us to obtain high resolution images for trapping analysis and cell stretching measurements. For smaller microchannels such as the fiber channel ($\approx$ 150 µm), the cleaning step to remove the residual wax is particularly important for easy insertion of the optical fibers and achieve optimal alignment. In our work, we managed to co-align the two fibers by manually inserting the fibers into the fiber channels while visualizing the process under a stereomicroscope. Particles down to $3 \;\mu \hbox {m}$ can be efficiently optically trapped using our device. Misalignment of the fibers cores with respect to each other will result to rotation of trapped particle in either X-Z or X-Y planes or both as observed in previous studies^37–39^. Despite the manual procedure of fiber insertion, rotation was not observed in our dual beam fiber traps and stable optical traps could be obtained.

Recent developments in two photon polymerization allowed fabrication of complex lenses^40^, microoptics^41^ to be incorporated at the tip of the fiber, as well as free form optics^42^, collectively demonstrating new approaches of optical trapping systems with enhanced trapping capabilities. Optical fibers could also be oriented on-chip in various configuration for multiple trapping, without the need for complex electro-optic devices^43^. 3D micromixers^5^ could potentially be combined with optical forces for sorting and analysis at high-throughput. Future works using 3D printing to produce microfluidic chips will allow more intricate designs that could permit novel combinations of fluidic and optical manipulation.

## Conclusion

In the present work, a fully 3D printed microfluidic device was presented for dual beam optical fiber experiments capable of capturing micrometer sized particles and manipulating mammalian cells. The microfluidic device can achieve stable optical trapping using a dual fiber beam geometry, which also enabled precise trap stiffness measurements. Mammalian cells can be successfully trapped and optically stretched, using this device which makes it possible to perform specific investigations, e.g. on the influence of chemical compounds or drugs on the elasticity of individual cells. We successfully demonstrated the effect of pyrichalasin H on MCF-7 cells. Overall, this paper demonstrates that current 3D printing technologies are able to fabricate robust microfluidic devices for optical manipulation. The advantages of 3D printing– such as rapid prototyping, reduced costs and comparatively easy integration of functional assemblies (e.g. optical fibers) can substantially simplify the overall process of developing and optimizing the design of customized microfluidic devices for a variety of applications.

## Materials and methods

### Microfluidic and test print fabrication

The microfluidic chip was fabricated using a high-definition Projet MJP2500 Plus multijet printer (3D Systems, Projet MJP2500 Plus, Rock Hill, USA). The printer was set to “high definition”, providing a layer resolution of $32\;\mu \hbox {m}$ and a print resolution of $800 \times 900$ dpi according to manufacturer. The printing material was VisiJet M2R-CL (3D Systems, Rock Hill, USA), which forms a solid polyacrylate upon UV exposure during the printing process. The proprietary material contains 3-Hydroxy-2,2-dimethylpropyl 3-hydroxy-2,2-dimethylpropionate diacrylate, Tricyclodecane dimethanol diacrylate, and the polymerization initiator Diphenyl(2,4,6-trimethylbenzoyl) phosphine oxide. The multijet printer used VisiJet M2 Sup support material (hydroxylated wax). After completion of the print, the devices were first cooled to 18${^\circ }$C for 10 min and then the support material was removed in two steps, first in a hot water vapor bath (60 $^{\circ }$C) and then in a mineral oil bath (3D Systems EasyClean units, Rock Hill, USA) bath. The microfluidic channels and test prints were sequentially flushed with mineral oil (EZRinse-C), 70% ethanol (Carl Roth GmbH und Co. KG, Karlsruhe, Germany) and deionized water, in order to remove any wax and oil residues. The test prints with features such as wells and protrusions shown in Fig. 1a,b were imaged and analyzed in 3D-panorama mode at 100$\times$ magnification using a 3D digital microscope (VHX-6000, Keyence Corporation, Osaka, Japan).

To clean the fiber channels and the circular hole above the trap region of the 3D printed device, a cleaning chip was designed as shown in the Figure S1. The cleaning chip applies pressure via fluid flow to the fiber channels to remove any residual wax. After flushing several times, the chip was dried overnight. A 12 mm microscopic cover slip was placed on the circular hole above the trapping region of the 3D printed device and the edges were sealed with a UV-curing optical adhesive (Norland Optical Adhesive, NOA68, Thorlabs, New Jersey, USA) cured for 30 minutes under a 365 nm, 6 W UV lamp (VL-6.LC, Vilber Lourmat GmbH). Two identical single mode optical fibers (SM980-5.8-125, Thorlabs, New Jersey, USA) with bare fiber at the distal end were used for the experiments. The jacket of the inserted fiber part was removed with a fiber stripper, then the bare end of the fiber was cleaned with 70% ethanol and terminated with a fiber cleaver (CL-03, UCL Swift, Daejeon, Korea). Both fibers were inserted into 3D printed device fiber channels from opposite sides until they reached the boundary of the flow channel. Finally, the UV-curable optical adhesive was carefully applied onto the insertion port of the fibers in the chip and the entire device was again exposed to a UV lamp for 20 min.

### Experimental setup

**Figure 6:**
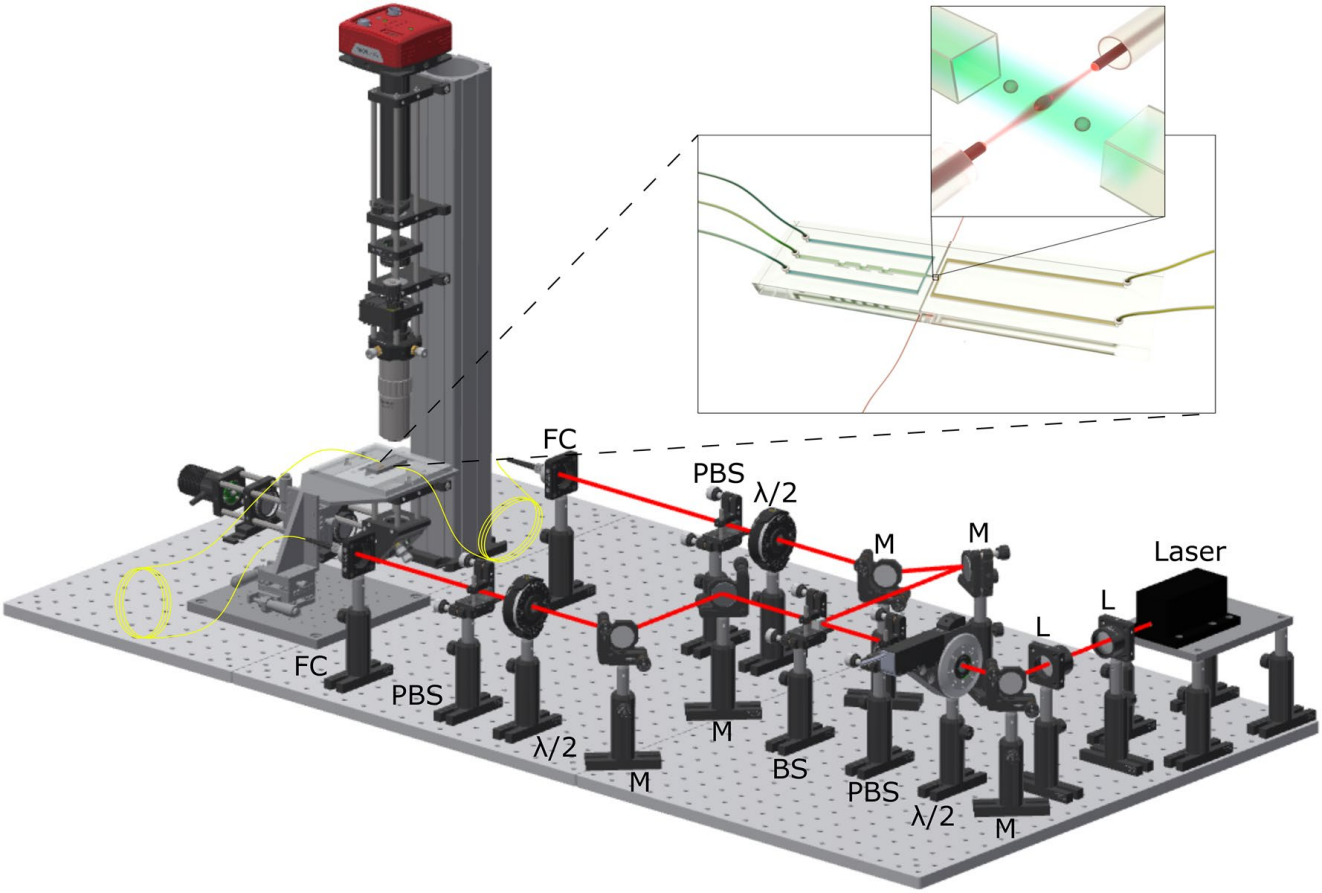
Setup of the dual beam optical trap experiment. The laser beam is divided into two beams with equal power and coupled into single mode fibers. The counterpropagating beams exiting the two optical fibers trap the particles and cells in the microfluidic channel. The dual beam optical trap is illuminated with a white light LED from the bottom side and imaged using a 50$\times$ long working distance objective onto a high speed CCD camera.

The schematic diagram of the trapping setup is shown in the Fig. 6. A 3 W diode pumped solid state, Nd:YVO$_4$ continuous wave laser (Smart laser system, Berlin, Germany) operated at a wavelength of $\lambda$ = 1064 nm was used for the trapping experiments. The laser beam was demagnified by a lens pair (L1 and L2) with focal lengths f1 = 75 mm and f2 = 25 mm and directed using a mirror (M) in the beam path into a 50:50 beamsplitter (BS) which splits the beam with equal power into two arms with equal power. Each arm delivered the laser into a fiber coupler (FC) which couples the beam into the single-mode optical fiber attached to the microfluidic chip. The optical path included three pairs of half-wave plate ($\lambda$/2) and polarizing beamsplitter (PBS), in order to control the total laser power and the power delivered into each arm.

Imaging was performed with a self-built microscope. A white light LED illuminates the sample via Köhler configuration. The position of the microfluidic chip was controlled by a precise X–Y–Z micrometer stage (Misumi, XYSG40, Tokyo, Japan). The field of view is magnified using a long working distance objective (50$\times$, NA = 0.55, WD =13 mm, Mitutoyo, Kawasaki, Japan) and a tube lens f$_{TL}$ = 200 mm projected the image onto a CCD camera (340M-USB, pixel size = $7.4\;\mu \hbox {m}$, Thorlabs, New Jersey, USA). The CCD camera can be operated with a maximum frame rate of 200.7 at full sensor operation.

### COMSOL simulations

The effect of channel design on the transport of particles through the microfluidic system was investigated using COMSOL Multiphysics 5.5 (COMSOL Inc., Stockholm, Sweden) as CFD simulation software. A straight channel and zig–zag channel with identical channel dimensions were compared. The simulation was divided into two parts, which were (1) a steady state simulation of the fluid flow, and (2) a time-dependent simulation of particles within the fully developed flow from (1). For the simulation of the steady state flow in (1), the dynamic viscosity and density of the fluid (cell culture medium) were set to 1050 mPa s and 1007 kg/m$^{3}$, respectively. The average inflow velocity with a laminar flow profile was set to 333 $\mu$m/s. For the simulation of the particle behavior in (2), 1000 particles were initialized at t = 0 s with a density proportional and velocity equal to the inflow velocity from (1). The particles used had a size of $20\;\mu \hbox {m}$ and a density of 1050 kg/m$^{3}$, in order to compare the results with mammalian cells. The forces acting on the particles were drag force (based on Stokes’ law) and gravity.

### Dual beam optical trapping of polystyrene particles

To characterize the performance of our trapping system, we trapped and measured the trap stiffness for polystyrene particles of different sizes: 1, 3, 5 and $10\;\mu \hbox {m}$ (PPS-1.0/3.0/5.0/10.0, refractive index n = 1.59, stock concentration=25 mg/ml, Kisker Biotech, Steinfurt, Germany). The particle containing stock solution was diluted with deionized water by a factor of 1:4000, 1:1000, 1:100 for 3, 5 and $10\;\mu \hbox {m}$ particles, respectively. The particle solutions were each transferred into a 5 ml Luer lock syringe connected to a precision microfluidic syringe pump (AL-1000, World Precision Instruments, Sarasota, USA) and flowed through the middle channel of the microfluidic device. Particles flowing in the region of sufficient light intensity are trapped at the center of the two counter-propagating beams. Trapped particles exhibit an overdamped Brownian motion under the control of the optical force. The laser power was controlled using a pair of polarizing beamsplitter (PBS) and half wave plate $(\lambda /2)$, with the orientation set by a motorized rotation mount. This allowed for a precise stepwise increase or decrease of the input laser power into the fiber. Laser powers (*P*) emitted from each fiber ranged from *P *= 34 mW to 255 mW. Initially, the particles were trapped at a relatively low laser power of *P *= 100 mW for 5 minutes in order to reduce the influence of the solution flow on the particle motion. Thereafter, the laser power was adjusted accordingly, and the time-varying particle position was recorded by the camera for 4 seconds.

The fluctuation of particle position under each power setting was recorded with a CCD camera using the following settings: 200 frames per second and 5 ms exposure time. To estimate the position of each particle per frame, edge detection was performed on the trapped particles using the Matlab function “Canny filter”. Then, the Hough circle transform was applied to predict the most probable position of the contour of the particle in each frame. Finally, the center of the particle contour was determined and saved for further analysis. Each step is illustrated in Figure S2 (a and b) and an example of the detected edge overlaid on an image of an optically trapped $10\;\mu \hbox {m}$ particle is shown in the Figure S2c.

### Optical stretching of human breast cancer cells

Michigan Cancer Foundation-7 (MCF-7), a human breast cancer cell line was used for the optical stretching experiments. Cells were cultured in Dulbecco’s Modified Eagle Medium (DMEM) Ham F-12 (Biochrom, 51448C, Berlin, Germany) supplemented with 10% Fetal bovine serum (FBS) and 1% penicillin/streptomycin solution (P/S) and maintained in 37$^{o}$C/5% CO$_{2}$ incubator. Cells were harvested at 90% confluency. For cell harvesting, the culture dish was first washed twice with 3 ml Dulbecco’s phosphate-buffered saline (DPBS) and incubated in 3 ml TrypLE (12604013, Thermo Fisher, Massachusetts, USA) at 37 $^{o}$C/5% CO$_{2}$ for 5 min. Afterwards, 7 ml fresh culture medium was added to the dish to deactivate the activity of TrypLE. 1 ml of the cell suspension was transferred into a new dish with 9 ml culture medium, for further cell cultivation, and the remaining cells were used for the experiments. The culture medium was changed every 4 days.

To determine the effects of pyrichalasin H, MCF-7 cells were incubated in complete medium with $50\,\upmu \mathrm{g}/\mathrm{ml}$ pyrichalasin H for 1 hour. Then, cells were dissociated and flowed through the middle channel of the microfluidic chip. Cell culture medium supplemented with $50\,\upmu \mathrm{g}/\mathrm{ml}$ pyrichalasin H was also flowed into the buffer channel. The stretching protocol was performed as follows: first, a single cell was trapped by the laser with a power of 200 mW emitted by each fiber. Once the cell was trapped stably, the laser power was reduced to 140 mW and maintained for 10 s. Then, the laser power was increased up to 500 mW within 2 s to stretch the cells. After 7 s, the laser power was reduced to 140 mW. All cell experiments followed the same protocol. The stretching process was recorded continuously with an exposure time of 15 ms at 66.7 fps.

Compared to the optically trapped particles, the cell boundaries were harder to visualize due to their poorer contrast with the imaging scheme used. Therefore, the contrast of the images was first enhanced, and the edges of the cell were extracted by Canny edge detection. The cell boundaries were further refined by connecting any gaps using morphological operations, dilation and erosion. An example of the detection process is shown in Figure S3.

## Supplementary Information

Supplementary Information.

## Data Availability

The data sets generated and/or analysed during the current study are available from the corresponding author on reasonable request.

## Code availability

Files such as Matlab scripts, sample data sets and STL files used in this work are available at 10.5281/zenodo.4687904.
