# Supplementary material for: 3D printed microfluidic lab-on-a-chip device for fiber-based dual beam optical manipulation
Source: Sci Rep. 2021 Jul 16;11:14584. doi: 10.1038/s41598-021-93205-9 (PMC8285473; doi:10.1038/s41598-021-93205-9)
Supplement: Supplementary file 1 — Supplementary Information. [file 41598_2021_93205_MOESM1_ESM.pdf]

Supporting Information

3D printed microfluidic lab-on-a-chip device for fiber-based dual beam optical manipulation

Haoran Wang, Anton Enders, John-Alexander Preuss, Janina Bahnemann, Alexander Heisterkamp, Maria Leilani Torres-Mapa

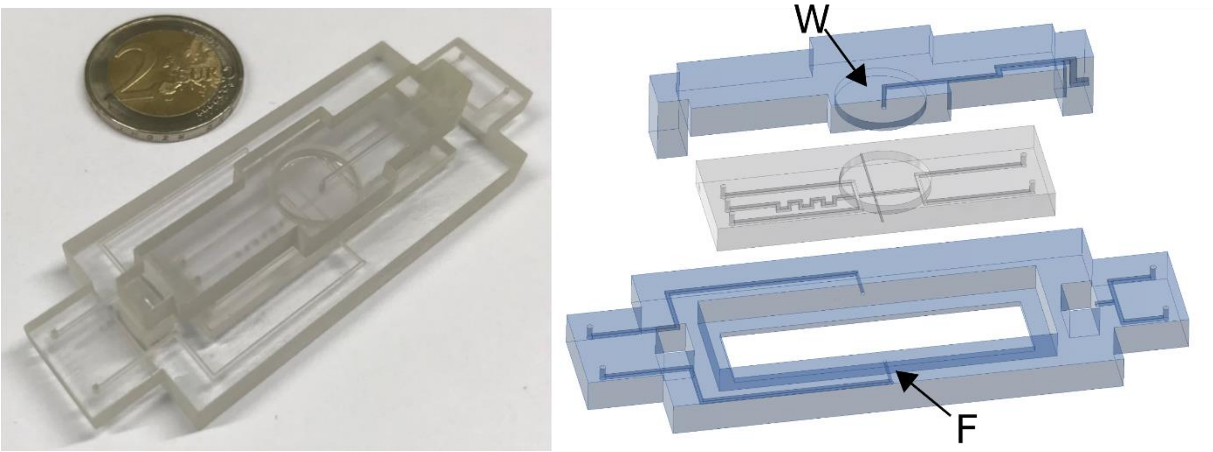

Figure S1. A cleaning chip is designed and used to effectively clean the fiber channels and the observation window of the 3D printed microfluidic device. Left image shows the actual photo of the chip. Right image shows the illustration of the assembly of the cleaning chip (blue) and the 3D printed microfluidic device (gray). Cleaning solution can be directly flushed into the fiber channel and the circular opening window connected to the channels of the cleaning chip, F and W, respectively. Using such a chip enable devices to have reproducible performance by removing any residual wax in the channels as well as the observation area.

The video data of the experiment were analyzed with self-written Matlab codes. Because of the difference in contrast as well as shape of the microscopic particles and mammalian cells, the image processing steps are distinct from each other as shown in Figure S2 and S3.

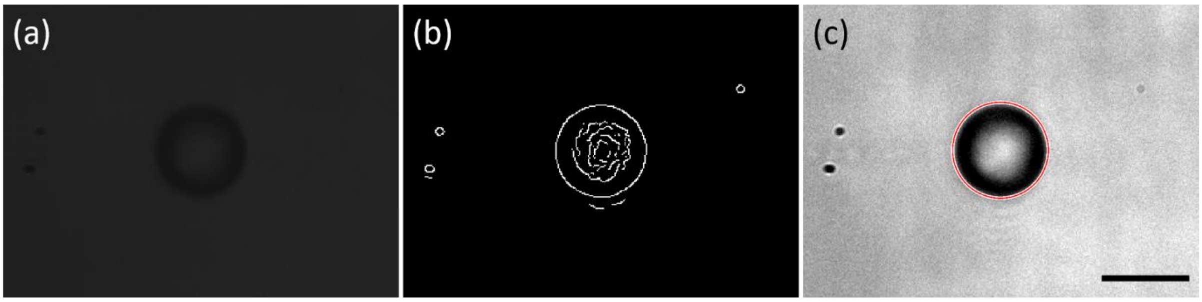

Figure S2. The image processing flow of the particle experiment data. (a) A single frame of the recorded video showing a 10 µm polystyrene particle trapped by the laser, (b) After changing the contrast, the contour of the particle is extracted by a Canny edge detection. (c) The position of the particle is calculated via Hough circle transform method. wherein the detected contour (marked with red) is overlayed on the particle. Scale bar is 10 µm.

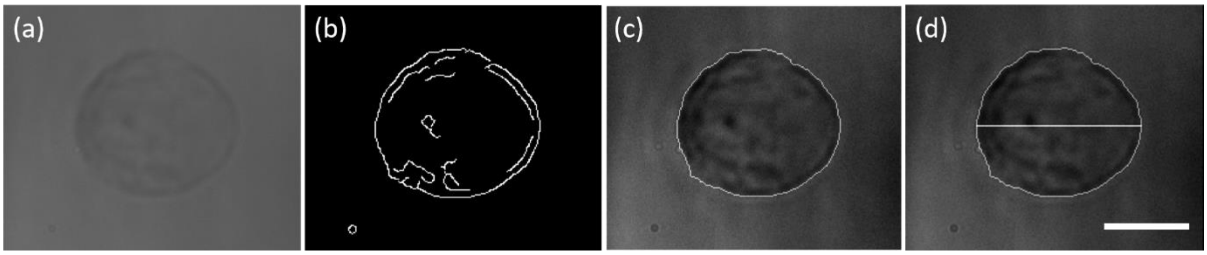

Figure S3. Flow of the image processing to determine the length of the cell during optical stretching. (a) The original image recorded by the camera. (b) The detected cell edge using the Canny edge detection. (c) After applying morphological methods such as erosion and dilation to the image, the small structures in the edge detection as seen in (b) are removed and the probable cell edge is connected with one single curve. (d) The cell length is derived by measuring the length of the cell at different regions within the cell boundaries. Thereafter, the maximum length is determined for every frame. Scale bar is 10 µm.
